# Mountainous millipedes in Vietnam. I. Two new species of the family Paradoxosomatidae from Mount Fansipan (Diplopoda, Polydesmida)

**DOI:** 10.3897/zookeys.1032.64917

**Published:** 2021-04-16

**Authors:** Anh D. Nguyen, Dai Dac Nguyen, Katsuyuki Eguchi

**Affiliations:** 1 Institute of Ecology and Biological Resources, Vietnam Academy of Science and Technology, 18, Hoangquocviet Rd., Caugiay District, Hanoi, Vietnam Institute of Ecology and Biological Resources, Vietnam Academy of Science and Technology Hanoi Vietnam; 2 Graduate University of Science and Technology, Vietnam Academy of Science and Technology, 18, Hoangquocviet Rd., Caugiay District, Hanoi, Vietnam Graduate University of Science and Technology Hanoi Vietnam; 3 Department of Biological Sciences, Faculty of Sciences, Tokyo Metropolitan University, Hachioji-shi, Tokyo Prefecture, Japan Tokyo Metropolitan University Tokyo Japan

**Keywords:** Biodiversity, *COI*, *16S rRNA*, new species, northwest Vietnam, taxonomy

## Abstract

Two new paradoxosomatid millipede species were discovered at the high elevations of Mount Fansipan in northwestern Vietnam. They are named as *Orthomorphoides sapa***sp. nov.** and *Hylomus solenophorus***sp. nov.** In addition to their morphological descriptions, sequences from fragments of two mitochondrial genes, *COI* and *16S rRNA*, are also provided for both new species.

## Introduction

At least three-quarters of Vietnam’s landscape is covered by mountains and hills (Le Ba Thao 2017). Not surprisingly then, this mountainous region is characterised by high biodiversity with many new species and genera being discovered annually (Sterling et al. 2006). Access to the high mountains is, however, very difficult resulting in limited biodiversity investigations, with millipedes from mountainous regions being particularly poorly known (Nguyen et al. 2019).

Among the high mountains in Vietnam, Mount Fansipan is very well-known as Vietnam’s highest peak, 3143 m a.s.l. It is located in the Hoang Lien Son Mountain Range, the southeasternmost extension of the Himalaya Range. The biodiversity of this mountain is very rich, containing both temperate and subtropical elements and both low- and highland species (Sterling et al. 2006). Little is known about the millipede fauna of Mount Fansipan. To date, only 23 species have been reported from this mountain and almost all of them are considered to be endemic to the region (Attems 1938, 1953; Golovatch 1984, 2009; Enghoff 1987; Golovatch and Enghoff 1993, 1994; Nguyen et al. 2005, 2019; Nguyen 2012) (Table 1).

**Table 1.** The known millipede species on Mt. Fanxipan, northwestern Vietnam.

Order	Family	Species
Glomerida	Glomeridae	*Hyleoglomeris fanxipan* Nguyen, Hwang & Eguchi, 2019
		*Hyleoglomeris sapa* Nguyen, Hwang & Eguchi, 2019
Sphaerotheriida	Zephroniidae	*Sphaerobelum separatum* (Attems, 1953)
Platydesmida	Andrognathidae	*Pseudodesmus camptotrichus* Attems, 1938
Polyzoniida	Siphonotidae	*Dawydoffia kalonota* Attems, 1953
Julida	Julidae	*Nepalmatoiulus fan* (Enghoff, 1987)
		*Nepalmatoiulus pan* (Enghoff, 1987)
Spirobolida	Spirobolellidae	*Physobolus annulatus* Attems, 1953
Chordeumatida	Kashmireumatidae	*Vieteuma topali* Golovatch, 1984
Polydesmida	Cryptodesmidae	*Trichopeltis kometis* (Attems, 1938)
Polydesmida	Paradoxosomatidae	*Chapanella rubida* Attems, 1953
		*Hylomus cervarius* (Attems, 1953)
		*Hylomus proximus* (Nguyen, Golovatch & Anichkin, 2005)
		*Kronopolites montanus* Golovatch, 2009
		*Oxidus gigas* (Attems, 1953)
		*Sapamorpha complexa* Golovatch, 2009
		*Sellanucheza variata* (Attems, 1953)
		*Tylopus crassipes* Golovatch, 1984
		*Tylopus magicus* Golovatch, 1984
		*Tylopus nodulipes* (Attems, 1953)
		*Tylopus provurcus* Golovatch, 1984
		*Tylopus sapaensis* Nguyen, 2012
		*Tylopus sigma* (Attems, 1953)

This work will contribute to a better understanding of the millipede fauna of Mount Fansipan by describing two new paradoxosomatid species found there. Both morphological and molecular data are provided for the new species.

## Material and methods

Material was collected from high elevations of Mount Fansipan, northwestern Vietnam, and preserved in 90% ethanol. Specimens were observed under an Olympus SZX10 microscope.

Images at various focal planes were taken under both normal and ultraviolet (UV) light using a micro-optics imaging system coupled with a Nikon D5100 camera (see Sierwald et al. 2019 for a detailed description of the UV imaging technique). Multiple images were processed in Adobe Lightroom, then stacked using Helicon Focus v. 4.0 and assembled using Adobe Photoshop CS6.

For the purposes of scanning electron micrography, gonopods were dissected, mounted on aluminium stubs, coated with gold and then studied using the LEO EVO 60 SEM system (Carl Zeiss) in the Field Museum of Natural History. After SEM imaging, the gonopods were returned and preserved with their specimen.

Total DNA was extracted from several midbody legs using the QIAGEN DNeasy Blood & Tissue Kit. Fragments of the mitochondrial *cytochrome c oxidase subunit I* (*COI*) and *16S rRNA* genes were amplified using two pairs of primers: COI-1F (5’-ACTCTACTAATCATAAGGAT-3’) and COI-1R (5’-TAAACCTCCGGGTGACCAA-3’), 16S-1F (5’-CCGGTTTGAACTCAGATCA-3’) and 16S-1R (5’-TGACTGTTTAGCAAAGACAT-3’). The amplification protocol followed a previously published method by Nguyen et al. (2017). Each successfully amplified and sequenced fragment was assembled using ChromasPro v. 2.1.8 and confirmed by BLAST searches (Zhang et al. 2000). All nucleotide sequences were deposited in GenBank.

All holotypes and paratypes were deposited in the Department of Soil Ecology, Institute of Ecology and Biological Resources, Vietnam Academy of Science and Technology, Hanoi, Vietnam.

## Results

### Taxonomy

#### Order Polydesmida Pocock, 1881

**Family Paradoxosomatidae Daday, 1889**

##### Genus *Orthomorphoides* Likhitrakarn, Golovatch & Panha, 2011

###### Orthomorphoides sapa sp. nov.

Taxon classificationAnimaliaPolydesmidaParadoxosomatidae

03FC3006-DAD2-51D4-BCDC-31BB9A8D9D55

http://zoobank.org/3BBAA752-06DC-47AD-894A-FAD9AD6E8283

**Figure 1.**
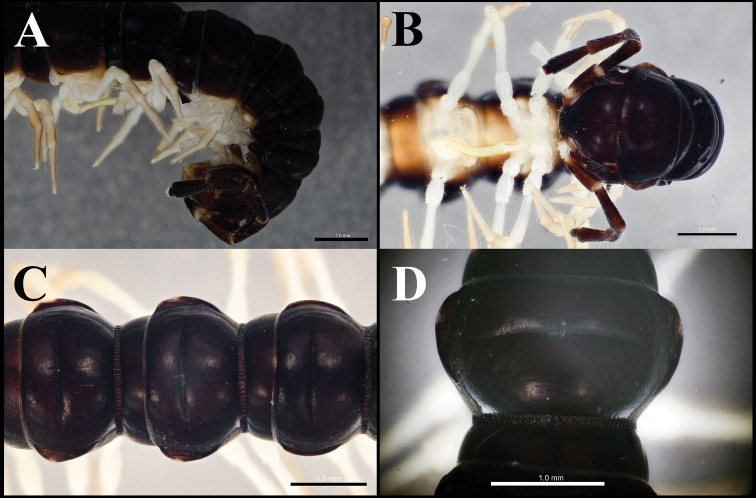
*Orthomorphoides sapa* sp. nov., holotype (IEBR-Myr 710H) anteriormost body segments, lateral view (**A**), ventral view (**B**) segments 8–10, dorsal view (**C**) segment 10, dorsal view (**D**).

**Figure 2.**
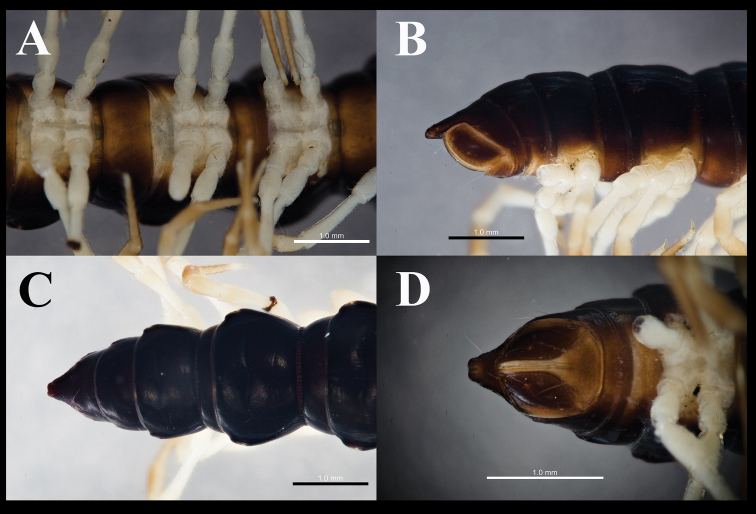
*Orthomorphoides sapa* sp. nov., holotype (IEBR-Myr 710H) body segments 8–10, ventral view (**A**) posteriormost segment, lateral view (**B**), dorsal view (**C**) telson, ventral view (**D**).

**Figure 3.**
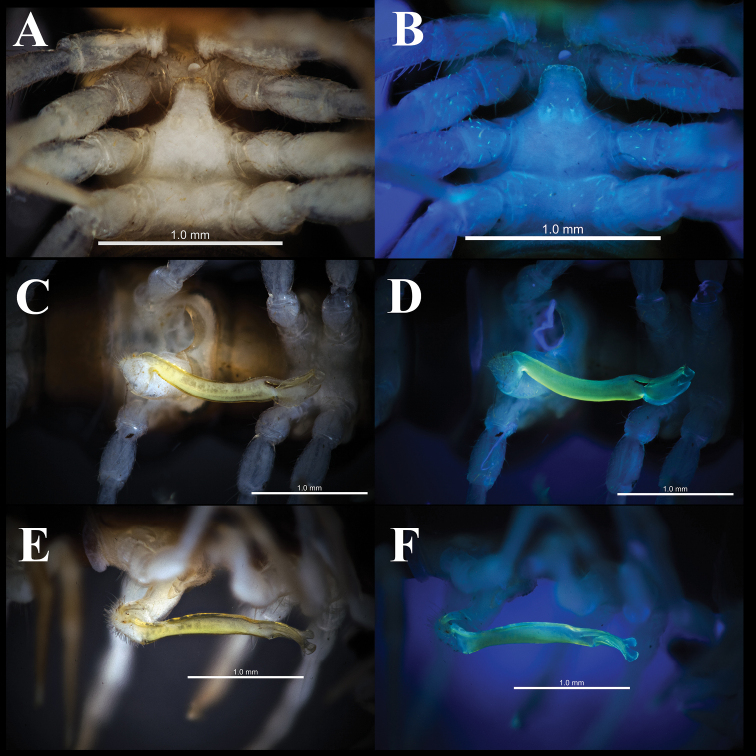
*Orthomorphoides sapa* sp. nov., holotype (IEBR-Myr 710H) sternal process between coxae 4, subventral view, normal light (**A**), UV light (**B**) right gonopod, ventral view, normal light (**C**), UV light (**D**), submesal view, normal light (**E**), UV light (**F**).

**Figure 4.**
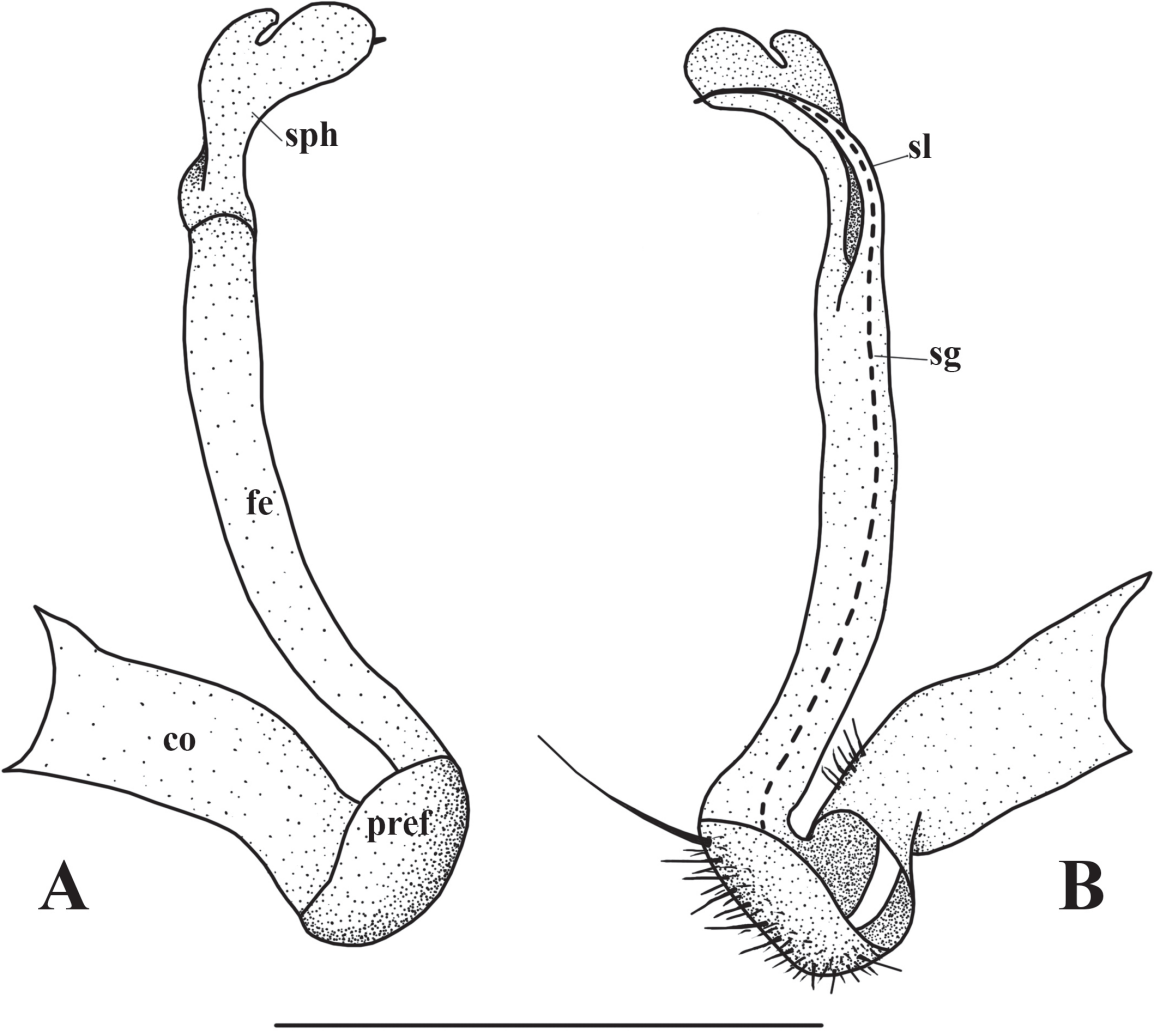
*Orthomorphoides sapa* sp. nov., holotype (IEBR-Myr 710H) **l**eft gonopod, lateral view (**A**), mesal view (**B**) co = coxite, pref = prefemorite, fe = femorite, sl = solenomere, sph = solenophore, sg = seminal groove. Scale bar: 0.5 mm.

**Figure 5.**
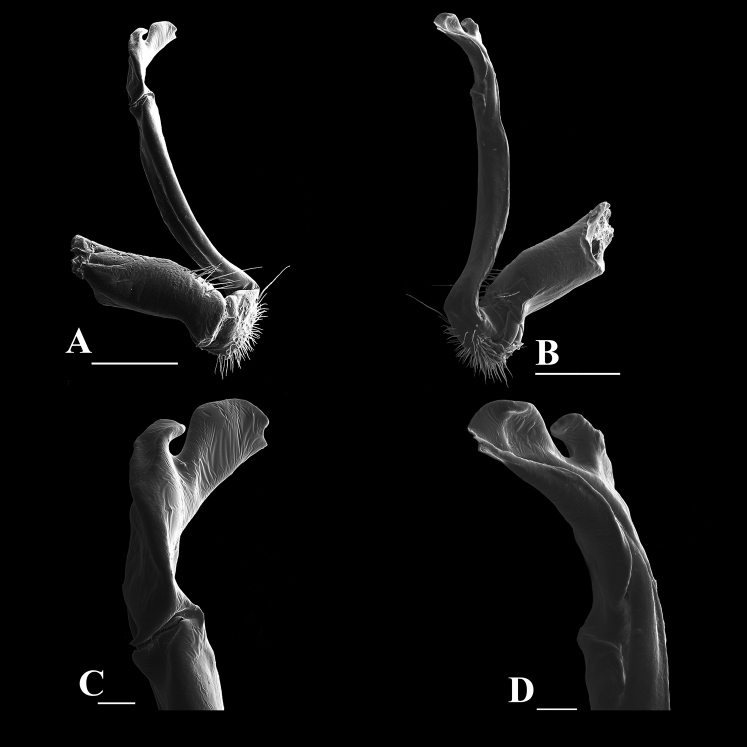
*Orthomorphoides sapa* sp. nov., holotype (IEBR-Myr 710H) left gonopod, lateral view (**A**), mesal view (**B**) postfemoral region of gonopod, lateral view (**C**), mesal view (**D**). Scale bars: 400 μm (**A, B**); 60 μm (**C, D**).

Figs 1 , 2 , 3 , 4 , 5

####### Material examined.

***Holotype*:** male (IEBR-Myr 710H), Vietnam, Lao Cai Province, Hoang Lien National Park, natural forest, 22.32250°N, 103.77081°E, 2478 m a.s.l., 7 July 2018, coll. Nguyen Dac-Dai. ***Paratype***: 1 female (IEBR-Myr 710P), together with holotype.

####### Diagnosis.

The species is easily recognized by having a black sub-moniliform body, small and crest-shaped paraterga, a long and slender gonofemorite, a simple solenophore with neither modifications nor additional processes, a poorly developed lamina medialis, a well-developed lamina lateralis with a tongue-shaped process, and the gonopod tip with a broad apical lobule.

####### Description.

Holotype body length about 22.1 mm, width of pro- and metazona 1.6 mm and 2.1 mm, respectively.

***Colouration*** (Figs 1–2): body black except whitish yellow legs and sterna; several podomeres light brown.

***Head*** (Fig. 1A, B) somewhat larger than collum, clypeolabral region densely setose, vertex sparsely setose. Epicranial suture distinct; frons with 2+2 setae along epicranial suture. Antenna long, slender, reaching segment 3 if stretched laterally; antennomere 2>3=4=5=6>1>7; tip with four sensory cones.

***Collum*** (Fig. 1B) semicircular; surface smooth, shining with two rows of setae: 4+4 anterior and 1+1 intermediate; transverse sulcus present, but short, indistinct. Paratergum present, crest-shaped.

***Body*** sub-moniliform (Figs 1C, D, 2C). Surface smooth and shining, without metatergal setae. Transverse metatergal sulci deep, line-shaped and present on all segments. Waist between pro- and metazonae relatively deep, striolate. Paraterga present, but small, crest-shaped, lying lower than metatergal surface. Pleurosternal carinae present as complete crests on segments 2–3, reduced to caudal teeth on segments 4–7, and missing on subsequent segments. Axial line missing.

***Epiproct*** (Fig. 2B–D) broadly truncated, flattened dorsoventrally; tip with four spinnerets. Hypoproct subtriangular, with two distolateral, separated setiferous knobs (Fig. 2D).

***Sterna*** sparsely setose, with distinct cross-impressions, without modifications except for a setiferous, broadly tongue-shaped lobule between coxae 4 (Fig. 3A, B).

***Legs*** slender, long about 1.6–1.8 times as long as midbody height. Femora without modifications. Prefemora not swollen. Tarsal brushes absent.

***Gonopods*** simple (Figs 3C–F, 4, 5). Coxite long, cylindrical, distoventral part sparsely setose. Prefemorite densely setose, set off from femorite by an oblique sulcus laterally. Femorite long, slender, without processes or modifications. The demarcation between postfemoral region and femorite present laterally. Lamina medialis of solenophore poorly developed while lamina lateralis well developed, with a tuberculiform process. Tip of gonopod broadly rounded lobule. Seminal groove running entirely on mesal side of femorite, then entering a flagelliform solenomere sheathed by solenophore.

####### DNA barcoding.

Fragments of *COI* and *16S rRNA* genes were uploaded to GenBank with the accession numbers: MW647898 and MW648327, respectively. The new species has a close *COI* gene similarity with *Orthomorphoides setosus* (KU234720) of 87.17%. It also shares 74.04% and 72.68% of its *16S rRNA* gene sequence with *Asiomorpha coarctata* (KU721885) and *Pogonosternum nigrovirgatum* (KU745218), respectively.

####### Etymology.

Named after Sapa, the type locality. It is a noun in apposition.

**Figure 6.**
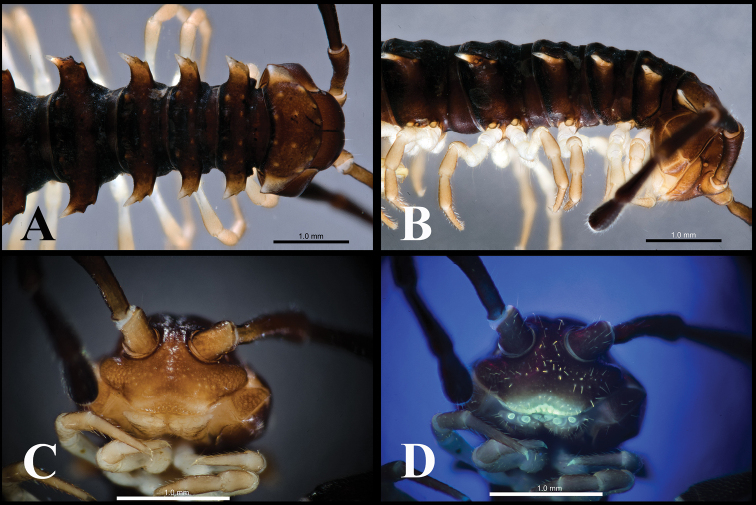
*Hylomus solenophorus* sp. nov., holotype (IEBR-Myr 712H) anteriormost body segments, dorsal view (**A**), lateral view (**B**) head, anterior view (**C, D**).

####### Remarks.

The genus *Orthomorphoides* was extracted from the genus *Orthomorpha* by Likhitrakarn et al. (2011) for two species: *Orthomorpha setosa* (Attems, 1937) and *Orthomorpha exarata* (Attems, 1953). The genus, therefore, currently contains only two species, *Orthomorphoides setosus* from Lam Dong Province (Vietnam) and *Orthomorphoides exaratus* from Xieng Khouang (Laos).

This new species obviously belongs to the genus *Orthomorphoides* because of its generic characters, such as: long and slender femorite without visible modifications and processes, and the solenomere being sheathed by a solenophore except for the exposed tip. The new species does, however, clearly differ from the two known *Orthomorphoides* species in body shape and the degree of development of the paraterga. In addition, the solenophore of the new species carries a tuberculiform process laterally while neither *O. setosus* nor *O. exaratus* possess additional processes on the solenophore.

The two previously described *Orthomorphoides* species were found only in high mountains in southcentral Vietnam and Xieng Khouang (>1500 m a.s.l.) (Attems 1953). The discovery of this new species, found in Sapa, may further support the mountainous distribution of this genus, although this must be confirmed with additional field studies and possible discoveries.

##### Genus *Hylomus* Cool & Loomis, 1924

###### Hylomus solenophorus sp. nov.

Taxon classificationAnimaliaPolydesmidaParadoxosomatidae

AE096862-6A33-5076-9359-79D831C62C2D

http://zoobank.org/2BAC98BA-8221-4C8B-B1B5-63ACD3534CE9

**Figure 7.**
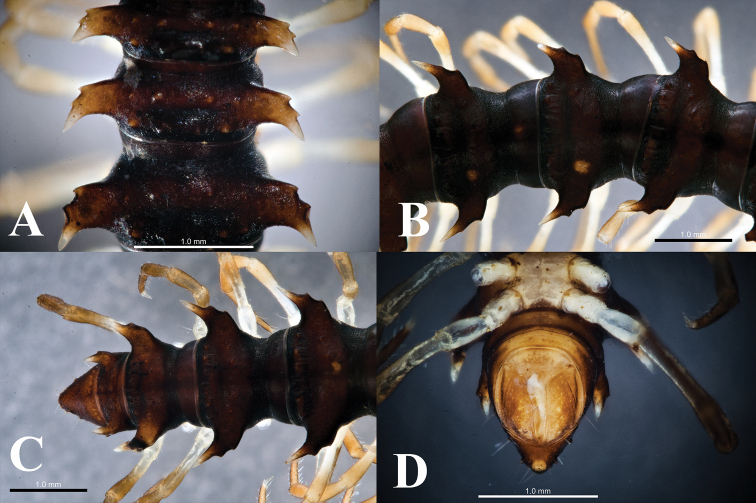
*Hylomus solenophorus* sp. nov., holotype (IEBR-Myr 712H) segments 8–10, dorsal view (**A, B**); posteriormost segments, dorsal view (**C**); telson, ventral view (**D**).

**Figure 8.**
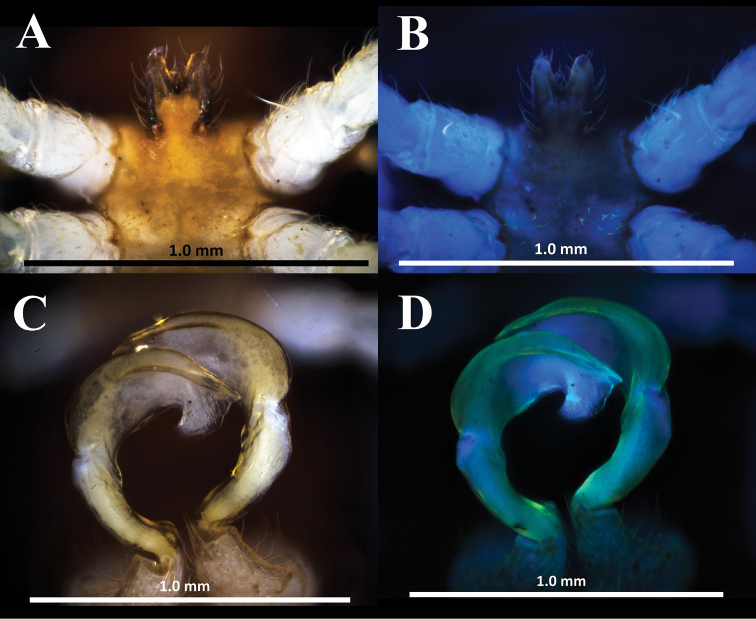
*Hylomus solenophorus* sp. nov., holotype (IEBR-Myr 712H) sternal process between coxae 4, subventral view, normal light (**A**), UV light (**B**) gonopods, ventral view, normal light (**C**), UV light (**D**).

**Figure 9.**
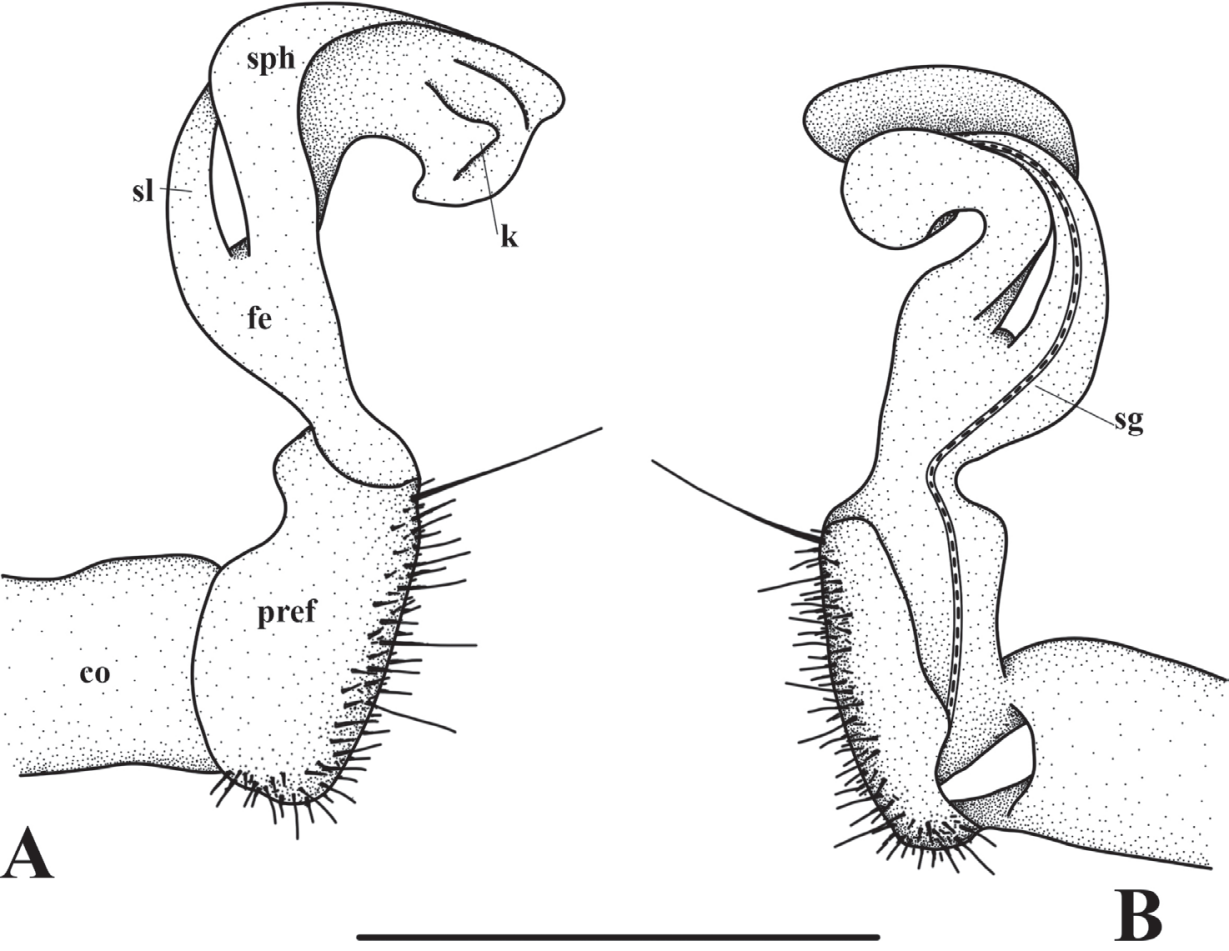
*Hylomus solenophorus* sp. nov., holotype (IEBR-Myr 712H) left gonopod, lateral view (**A**), mesal view (**B**) co = coxite, pref = prefemorite, fe = femorite, sl = solenomere, sph = solenophore, sg = seminal groove, k = distoapical process k. Scale bar: 1 mm.

**Figure 10.**
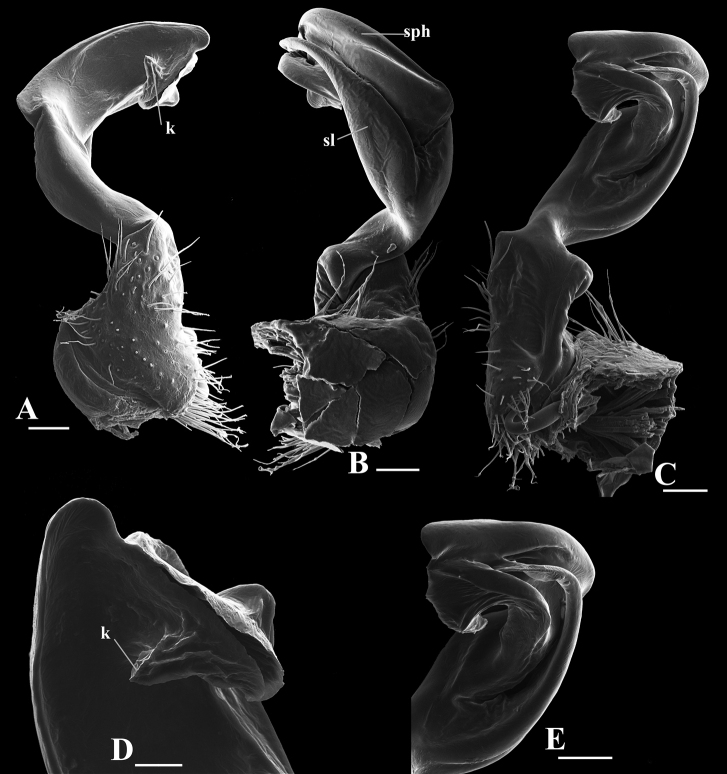
*Hylomus solenophorus* sp. nov., holotype (IEBR-Myr 712H) left gonopod, ventral view (**A**), lateral view (**B**), mesal view (**C**) postfemoral region, mesal view (**D**) tip of gonopod, ventral view (**E**) sl = solenomere, sph = solenophore, k = distoapical process k. Scale bars: 100 μm (**A–C, E**); 40 μm (**D**).

Figs 6 , 7 , 8 , 9 , 10

####### Material examined.

***Holotype*.** male (IEBR-Myr 712), Vietnam, Lao Cai Province, Hoang Lien National Park, natural forest, 22.32250°N, -103.77081°E, 2478 m a.s.l., 7 July 2018, coll. Nguyen Dac-Dai. ***Paratype*.** 1 male, 1 female (IEBR-Myr 714), Lao Cai Province, Hoang Lien National Park, natural forest, 22.32129°N, -103.77094°E, 2547 m a.s.l., 7 July 2018, coll. Nguyen Dac-Dai.

####### Diagnosis.

The species differs from its congeners by having wing paraterga, two rows of 2+2 and 2+2 knobs on the metaterga, and a well-developed, broad gonopod solenophore with an additional distal process.

####### Description.

Holotype body length about 16.0 mm, width of pro- and metazona 1.2 mm and 2.0 mm, respectively.

***Coloration*** (Figs 6, 7): whole body blackish brown or black, except labrum, sterna and legs yellowish brown. Projected caudal corners of paraterga yellowish brown.

***Head*** (Fig. 6C, D) slightly smaller than body segments, but somewhat larger than collum. Epicranial suture clearly distinct; frons with 2+2 setae along epicranial suture. Clypeolabral region moderately setose. Antenna extremely long, reaching segment 5 if stretched laterally; antennomere 3=4=5>2>6>1>7; tip with four sensory cones.

***Collum*** (Fig. 6A) slightly smaller than segment 2, semicircular; surface not smooth, with 3 rows of setiferous knobs: 3+3 anterior, 2+2 intermediate and 1+1 posterior; transverse sulcus present, located near a row of 1+1 posterior knobs. Paratergum broadly triangular, well developed, with a lateral incision (Fig. 6A).

***Body*** segment 3<4<2=5–17, thereafter gradually tapering toward telson (Figs 6A, B, 7A–C). Prozonae smooth, shagreened while metazonae densely covered with microgranulation. Transverse metatergal sulci shallow, broad, present on all segment. Metaterga 2–4 with two rows of setiferous knobs: 2+2 and 2+2 in front of and behind transverse sulcus, respectively; other metaterga with two rows of 2+2 and 3+3 setiferous knobs. Axial line distinct, thin. Waist between pro- and metazonae indistinct, shallow, and broad. Pleurosternal carinae absent.

***Paraterga*** (Figs 6A, 7A–C)) wing-shaped, with pointed, projected caudal corners, lying horizonally, but reduced as broad-base spine with two lateral incisions on segments 7–18.

***Epiproct*** long, broadly truncated, flattened dorsoventrally; tip with four spinnerets (Fig. 7D). Hypoproct trapeziform, with two distolateral, separated setiferous knobs (Fig. 7D).

***Sterna*** sparsely setose, with distinct cross-impressions, without modifications except for an anterioventrally directed, large, strongly bi-lobuled process between coxae 4 (Fig. 8A, B).

***Legs*** slender, long about 1.5–1.7 times as long as midbody height. Prefemora not swollen. Femora without modifications. Tarsal brushes absent.

***Gonopods*** simple (Figs 8C, D, 9, 10). Coxite cylindrical, distoventral part sparsely setose. Prefemorite densely setose, set off from femorite by an oblique sulcus laterally. Femorite slightly curved mesad, somewhat enlarged distally, without modifications and processes. Demarcation between femorite and postfemoral region absent. Postfemoral region consisting of solenophore and solenomere. Femorite and solenophore subequal in length. Solenophore simple, broad, slightly spiral and somewhat curved down. Tip of gonopod broadly round, with an additional distoapical process. Seminal groove running entirely on mesal side of femorite, then entering the flagelliform solenomere sheathed by solenophore.

####### DNA characters.

Fragments of COI and 16S rRNA were uploaded to GenBank with accession numbers: MW647899 and MW648328, respectively. The new species has a close COI identity with *Hylomus cervarius* (MG669370) and *Hylomus enghoffi* (MG669369) of 85.1% and 83.45%, respectively. It also shares 79.57% and 78.72% of its *16S rRNA* gene sequence with *Hylomus cervarius* (MG564329) and *Hylomus enghoffi* (MG564330), respectively.

####### Etymology.

An epithet “*solenophorus*” is used to emphasize the well-developed, broad solenophore carrying a triangular distoapical process.

####### Remarks.

The genus *Hylomus* Cook & Loomis, 1924 used to be a synonym of the genus *Desmoxytes* Chamberlin, 1923 (Golovatch and Enghoff 1994), but it was recently re-evaluated by Srisonchai et al. (2018) and currently comprises 39 species (Sierwald and Spelda 2021). The new species differs distinctly from the majority of known *Hylomus* species (except for *H. spectabilis*) in having wing-shaped paraterga (vs. antler-like or spiniform paraterga). As already stated, *Hylomus solenophorus* sp. nov. is closely similar to *H. spectabilis* (Attems, 1937) from central Vietnam by having wing-shaped paraterga and the same gonopod conformation. On the other hand, the newly described species differs clearly from *H. spectabilis* by being smaller in size (length: 16 mm vs. 30 mm; width of metazona: 2.0 mm vs. 6.1 mm) and having darker body coloration (darkish brown or black vs. brown). Furthermore, leg femora of the new species possess no visible modifications, metaterga contain two rows of 2 + 2 and 3 + 3 setiferous knobs and the gonopod has no spine z, but possesses a triangular distoapical process, instead. By comparison, the 7^th^ femur of the *H. spectabilis* male has a ventral hump, metaterga are characterised by two transversal rows of 2 + 2 well-developed spines and 1 + 1 smaller spines and the gonopod is characterised by well-developed spine z, but no process k.

## Discussion

The Hoang Lien Son Mountain Range with Mt. Fansipan plays a very important role in general geodistribution of animals in Vietnam, particularly of millipedes (Sterling et al. 2006). This mountain range is considered the southeasternmost extension of the Himalaya Range containing both Indian and Chinese zoological features. The Hoang Lien Son granites date from 80 to 29 million years ago, while the uplift of the range by tectonic activity began around 65 million years ago and continues to this day. This is also an interesting place for both ecological and biodiversity studies (Sterling et al. 2006). Despite its important role, the fauna of the mountain, in particular, the soil invertebrates, is still poorly known.

As described by Attems (1937, 1953), Enghoff (1987), Golovatch (1984, 2009), Nguyen et al. (2005) and Nguyen (2012), few millipede species have been discovered in this mountain. The recent record of two new species, *Hyleoglomeris hoanglien* (Nguyen, Eguchi & Hwang, 2019) and *Hyleoglomeris fanxipan* (Nguyen, Eguchi & Hwang, 2019) at very high elevation of 2800 m, proves that this region needs to be more intensively surveyed in order to obtain a comprehensive knowledge of the local fauna (Nguyen et al. 2019).

The Paradoxosomatidae is a huge family containing more than 1000 species distributed in about 220 genera (Nguyen and Sierwald 2013). Phylogenetic relationships between the paradoxosomatid genera and species within the family are still questionable. There are, however, some available DNA data for members of this family (183 *COI* gene records and 53 *16S rRNA* gene records in GenBank). It is not considered very practical to analyse the relationships between the two new species (belonging to different genera) and other known paradoxosomatid species using two short fragments of *COI* and *16S rRNA* genes. As a result, although the DNA barcoding data are provided here for the two new species, neither the genetic divergence nor phylogenetic relationships are taken into account. The provided DNA barcoding data should be considered an additional characterisation tool for the new species descriptions and identification.

## Conclusion

Most of World’s high-mountain regions are very difficult to access, but their natural habitats are well conserved. It is, therefore, highly expected that more intensive surveys in those regions will reveal many new taxa, not only of millipedes but also of other invertebrates.

## Supplementary Material

XML Treatment for Orthomorphoides sapa

XML Treatment for Hylomus solenophorus
